# A Novel Fungal Hyperparasite of *Puccinia striiformis* f. sp. *tritici*, the Causal Agent of Wheat Stripe Rust

**DOI:** 10.1371/journal.pone.0111484

**Published:** 2014-11-04

**Authors:** Gangming Zhan, Yuan Tian, Fuping Wang, Xianming Chen, Jun Guo, Min Jiao, Lili Huang, Zhensheng Kang

**Affiliations:** 1 State Key Laboratory of Crop Stress Biology for Arid Areas and College of Plant Protection, Northwest A&F University, Yangling, Shaanxi 712100, P. R. China; 2 USDA-ARS, Wheat Genetics, Physiology, Quality, and Disease Research Unit, and Department of Plant Pathology, Washington State University, Pullman, WA 99164–6430, United States of America; Korea University, Republic of Korea

## Abstract

*Puccinia striiformis* f. sp. *tritici* (*Pst*), the causal fungus of wheat stripe rust, was previously reported to be infected by *Lecanicillium lecanii*, *Microdochium nivale* and *Typhula idahoensis*. Here, we report a novel hyperparasite on *Pst*. This hyperparasitic fungus was identified as *Cladosporium cladosporioides* (Fresen.) GA de Vries based on morphological characteristics observed by light and scanning electron microscopy together with molecular data. The hyperparasite reduced the production and viability of urediniospores and, therefore, could potentially be used for biological control of wheat stripe rust.

## Introduction

Wheat stripe rust is one of the most destructive diseases of wheat worldwide [1]. It causes huge yield losses every year in China and many other countries [2]. *Puccinia striiformis* f. sp. *tritici* (*Pst*), the causal agent of wheat stripe rust, which is an obligate biotrophic fungus, normally forms yellow to orange urediniospores during disease progression. During greenhouse multiplications of *Pst*, the uredinia sometimes gradually become dark gray, especially under high humidity. At the beginning of sporulation, only few uredinia change from light gray to gray. However, more and more uredinia darken over time. Eventually, all uredinia become dark gray and no longer produce urediniospores. This discoloration and cessation of spore production may be associated with infection by a hyperparasite.

It is well known that plant pathogenic fungi can be infected by mycoparasitic fungi [3], [4]. For example, *C*. *cladosporioides* has been reported to be very effective in preventing growth and sporulation of the apple scab fungus (*Venturia inaequalis*) [5]. The species also exhibits antifungal activity against *Phomopsis viticola*, *Colletotrichum acutatum*, *C. fragariae* and *C. gloeosporioides* [6]. Approximately 30 genera of fungi can be hyperparasitic on rust fungi [7]. Among these, several *Cladosporium* spp., such as *C*. *aecidiicola*, *C*. *sphaerospermum* and *C. uredinicola* have been reported to parasitize fungi of the order Pucciniales [8], [9], [10]. However, only three species, *Lecanicillium lecanii*, *Microdochium nivale* and *Typhula idahoensis* have been reported to infect uredinia and urediniospores of *Pst* [7].

In the present study, we identified a novel hyperparasite of *Pst*. Based on morphological characteristics and molecular data, the fungus was identified as *Cladosporium cladosporioides* (Fresen.) GA de Vries, which has not been reported on *Pst* previously. Infection by *C. cladosporioides* reduced *Pst* urediniospore production and viability. Our results indicate that the *C. cladosporioides* strain identified may be effective in the biological control of stripe rust.

## Results

### SEM observations of *Pst* infected by the parasitic fungus

**Figure 1:**
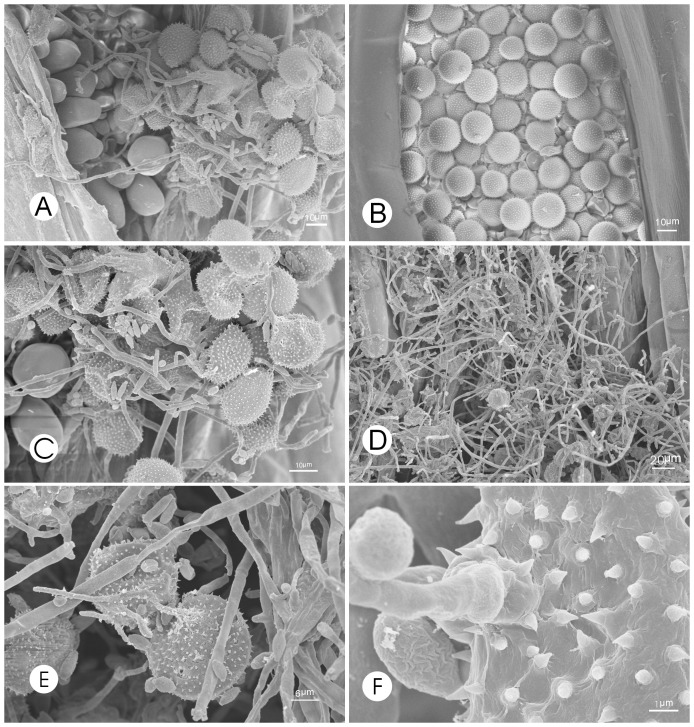
Uredinia and urediniospores of *Pst* are parasitized by *Cladosporium cladosporioides*. **A**: A uredinium infected by *C*. *cladosporioides* (×800), **B**: A non-infected uredinium filled with urediniospores (×700), **C**: Hyphae of *C. cladosporioides* growing from or between urediniospores at an early stage of infection (×2,000), note the shriveled urediniospores, **D**: Numerous hyphae of *C. cladosporioides* growing on a uredinium at a late stage of infection (×400), note presence of very few normal urediniospores, **E**: Urediniospores invaded by *C. cladosporioides* hyphae (×1,800), **F**: High magnification of a *C. cladosporioides* hypha invading a urediniospore (×11,000).

Observations with a scanning electron microscope (SEM) revealed that fungal hyphae grew extensively on the surface of a *Pst* uredinium, previously identified to have a light gray color. By contrast, a normal yellow-colored uredinium was devoid of visible hyphae growing on its surface (Figure 1 A, B). When a uredinium became dark gray, gray hyphae grew from urediniospores, indicating that they had been parasitized by a filamentous fungus. The morphology of these infected urediniospores changed markedly. The spore walls ruptured or were depressed inward (Figure 1 C–F), suggesting that the infected urediniospores lost the ability to germinate.

### Morphological observations by light and electron microscopy

**Figure 2:**
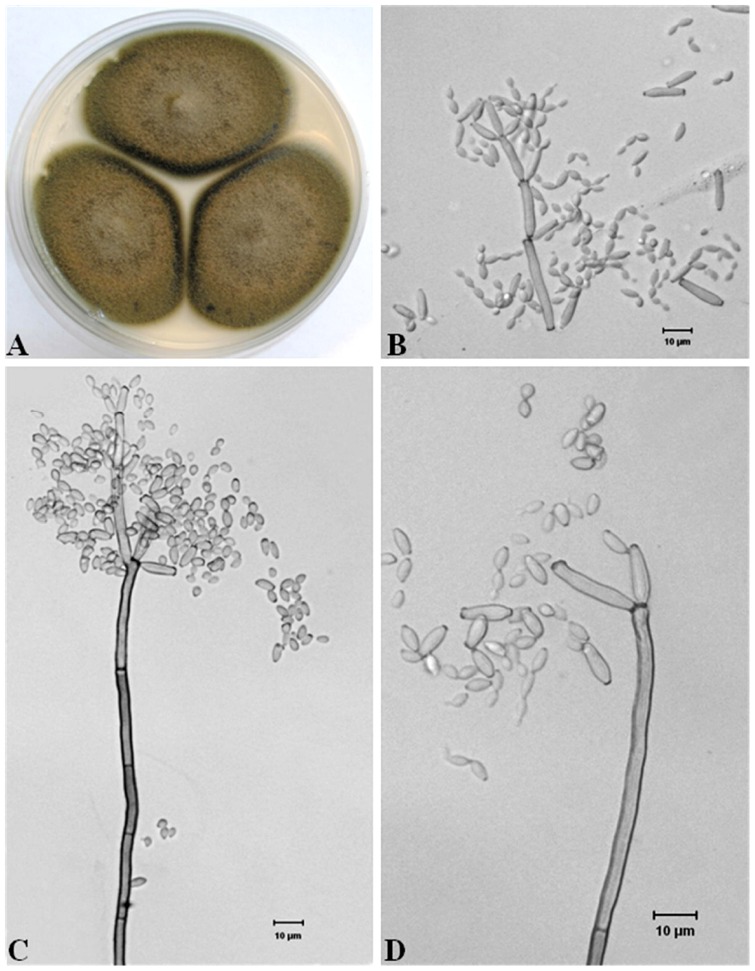
Morphological features of *Cladosporium cladosporioides* cultured on MEA medium. **A**: Fungal colonies on MEA medium, **B**: Branched ramoconidia and chains of conidia, **C**: Ramoconidia and dense conidia on the tip of a conidiophore, **D**: Oval to spherical conidia and separated ramoconidia with a dark scar at one end or both ends.

**Figure 3:**
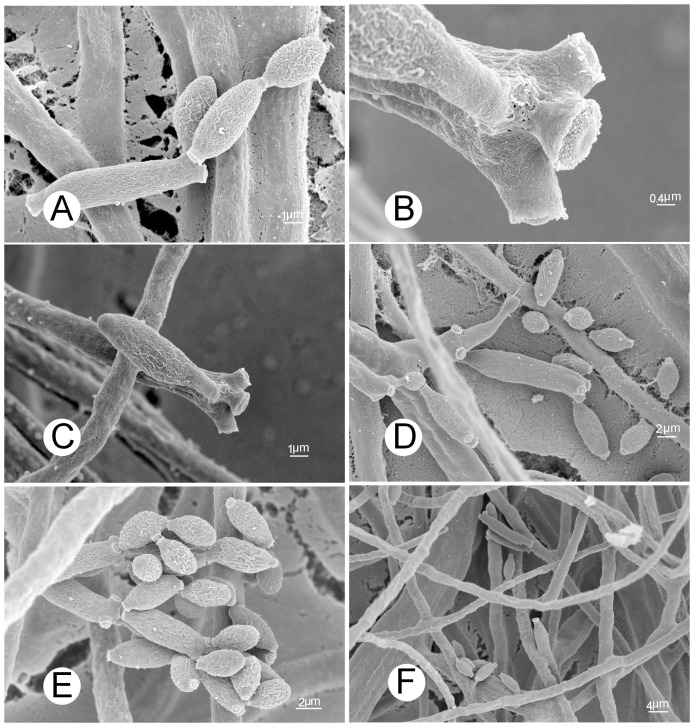
Morphological features of *Cladosporium cladosporioides* under a scanning electron microscope. **A**: Conidial chains (×7,000), **B**: Scars on a conidiophore (×20,000), **C**: Top view of a conidiophore with scars (×7,000), **D**: scars on a secondary ramoconidium (×4,000), **E**: Conidia on conidiophores (×5,000), **F**: Hyphae (×2,000).

The hyperparasitic fungus isolated from *Pst* uredinia grew rapidly on malt extract agar (MEA) medium, forming olive-gray to olive-green colonies with gray aerial mycelia (Figure 2A). By light microscopy, hyphae were found to be unbranched or only occasionally branched, and 1–4 µm wide. In branched hyphae, the branch sites were not associated with swollen or constricted regions. Conidiophores were solitary, straight or slightly curved, without nodules, and occasionally geniculate, 40–300 µm×3–4 µm. They were unbranched or occasionally branched, had a tapering tip, and the base was sometimes swollen (Figure 2 B–D). Ramoconidia were usually produced in groups of three at the tips of conidiophores. They were long and cylindrical (12–55 µm×3–5 µm), straight or slightly curved with a fine toothed projection. Conidia were formed in chains, nearly spherical, oval or lemon-shaped, 3–6 µm×2–3 µm. The more detailed morphological characteristics of conidia, ramoconidia, conidiophores and mycelia obtained by observations with a SEM are illustrated in Figure 3 A–F. Based on the morphological characteristics, the filamentous fungus was identified as *Cladosporium cladosporioides* (Fresen.) GA de Vries. The *C. cladosporioides* culture was deposited in China General Microbiological Culture Collection Center (CGMCC), Beijing, China (http://www.cgmcc.net) with an accession number of CGMCC 7.175.

### Confirmation of the hyperparasitic phenomenon

**Figure 4:**
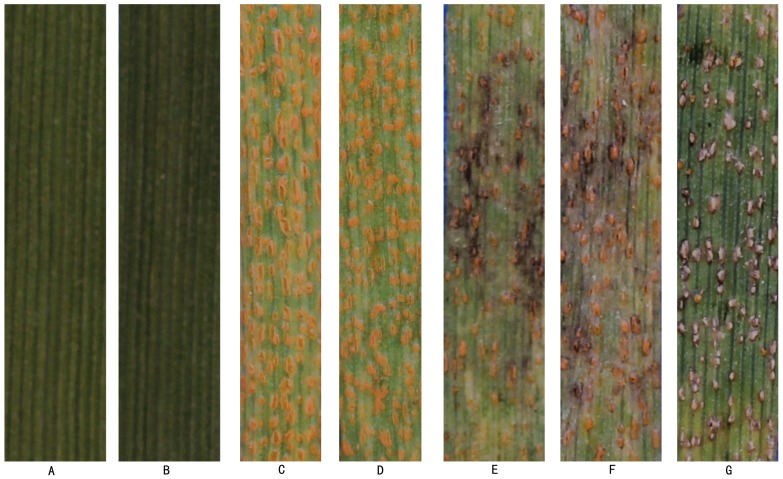
The pathogenicity test to confirm that *C. cladosporioides* could hyperparasite *Pst*. **A**, **B**: CK1, wheat leaves (Cultivar: MingXian169) inoculated with *C. cladosporioides*, 14 dpi, without symptoms; **C**, **D**: CK2, Symptoms on wheat leaves (MingXian169) inoculated with CYR32, 20 dpi; **E**, **F**, **G**: Pustules on wheat leaves (same cultivar and race as above) inoculated at 14 dpi with *C. cladosporioides* suspension. Symptoms at 5d, 7d and 9d later.

**Figure 5:**
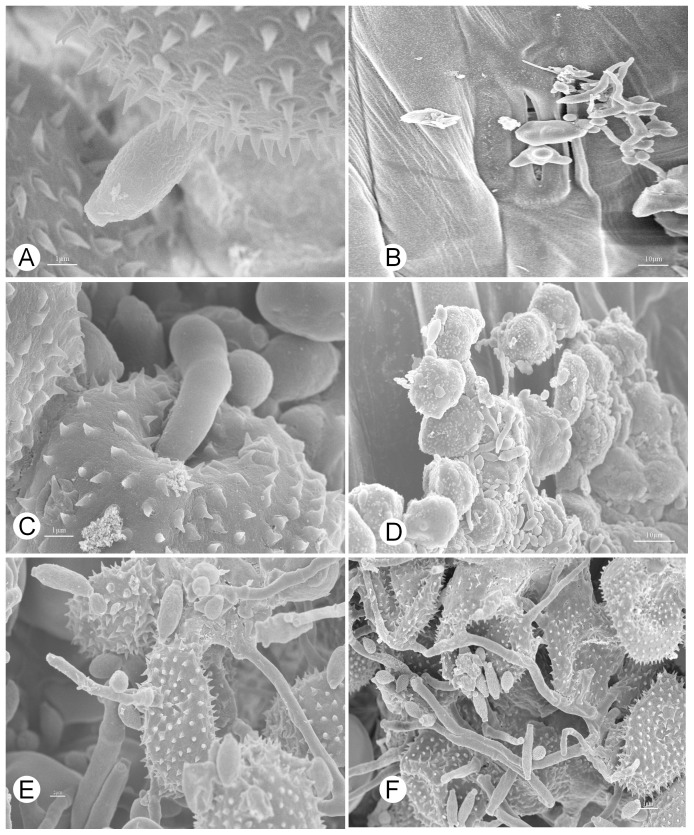
SEM observations of *Pst* inoculated with *C*. *cladosporioides*. **A**: 12 h after inoculation, the spore of *C*. *cladosporioides* contacted the urediniospore surface (×7,000), **B**: 24 h after inoculation, spores of *C*. *cladosporioides* germinated (×700), **C**: 36 h after inoculation, direct penetration by germ tube of *C*. *cladosporioides* into a urediniospore (×10,000), **D**: 48 h after inoculation, amount of urediniospores were surrounded with germinated spores of *C*. *cladosporioides* (×2,000), **E**: 72 h after inoculation, germ tubes of *C*. *cladosporioides* have penetrated urediniospores (×3,000), **F**: 120 h after inoculation, *C*. *cladosporioides* has colonized the urediniospores (×1,300).

No *C. cladosporioides* conidia or hyphae were observed on inoculated leaf surfaces in areas devoid of *Pst* uredinia (Figure 4A, 4B). Plants inoculated with only *Pst* urediniospores produced normal-colored uredinia and normal quantities of urediniospores, without any observed gray hyphae (Figure 4C, 4D). In contrast, the change in uredinial color, gray mycelial growth on uredia, and reduction of urediniospore production were observed on leaves 5–9 days after inoculation with the mycoparasite (Figure 4E, 4F, 4G). A pure culture obtained from the inoculated diseased leaves exhibited the same morphological characteristics as the organism in Figure 2A. The results confirmed that the *C. cladosporioides* strain parasitizes *Pst*. SEM observations of *Pst* inoculated with *C*. *cladosporioides* showed that spores of *C*. *cladosporioides* contacted the surface of urediniospore, germinated and the germ tubes directly penetrated urediniospores. After successfully invading and colonizing the urediniospores, the hyperparasite likely absorbed nutrition from urediniospores, causing them to appear collapsed and lose viability (Figure 5A–F).

### Sequence analysis of *C. cladosporioides* ITS, ACT and EF1-α

**Figure 6:**
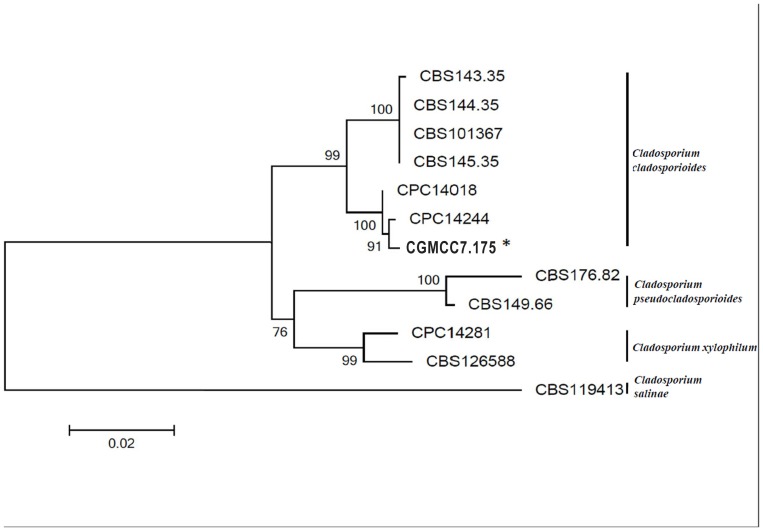
A neighbor-joining tree constructed using the sequences of ITS,EF and ACT to determine the phylogenetic relationship of the hyperparasitic fungal strain CGMCC 7.175 (bold and marked with *) isolated from uredinia of *Pst* with other accessions of *Cladosporium* spp. in GenBank. The values at braches are percentages from 1,000 bootstrap replications.

The genomic sequences of internal transcribed spacer (ITS) region, actin (ACT) and translation elongation factor 1-α (EF) supported the identification of the fungal strain as *C. cladosporioides*. Phylogenetic analysis with these sequences (GenBank accession numbers: KJ598781 for ITS, KM281944 for ACT and KM281945 for EF) clustered the fungus and eleven other known *Cladosporium* spp. into four groups, and CGMCC 7.175 clustered with six other isolates of *C. cladosporioides* in accordance with combined ITS, ACT and EF sequences (Figure 6). The *C. cladosporioides* group was divided into two subclades. CBS143.35, CBS144.35, CBS145.35 and CBS101367 formed one subclade, while CPC14018, CPC14244 and KJ598781 formed the other. Our novel strain (CGMCC 7.175) was found to be most closely related to CPC14244.

## Discussion

Spore size is an important parameter in morphological identification of species within the genus *Cladosporium*. However, many of the spore dimensions overlap among species in this genus, making it difficult to identify different *Cladosporium* species [11], [12]. ITS sequencing is also an effective approach for fungal species identification [13]. However, ITS sequences of *Cladosporium* spp. often exhibit a high degree of similarity among species, which makes it difficult to distinguish them. Dugan et al. [14] used three conserved genes, ITS, EF1-α and ACT, in a multi-gene sequence alignment to effectively distinguish *C*. *sphaerospermum* from other species in the genus. In the present study, we used an approach combining morphological characterization and multi-gene sequence alignment to determine the species of the hyperparasitic fungal strain as *C. cladosporioides*. *C. cladosporioides* is widely distributed, grows rapidly, and is well adapted to various environments [15], [16], [17]. By comparing with previously reported mycoparasites, the fungus identified in the present study is a new species parasitizing *Pst*. It is unclear if this particular strain can grow on other natural substrates beyond *Pst* uredinia.

Current practical control of wheat stripe rust is focused mainly on resistance breeding and fungicides. The rapid loss of race-specific resistance in wheat cultivars and potential resistance of the pathogen to fungicides are enormous challenges to develop strategies to minimize damage of wheat from stripe rust. There have been few attempts to control *Pst* with hyperparasites [7]. Fungi in genus *Cladosporium* have been previously reported to be effective in biocontrol of *Melampsora* spp. [18]. The *C. cladosporioides* strain discovered in the present study is a new hyperparasite to *Pst*. We observed that infection by the hyperparasite reduced urediniospore production and viability, and therefore, it may provide a new option for stripe rust management using a biological control approach. Based on our study, the strain is easily grown on artificial media.

Preservation of *Pst* urediniospores is important for studying wheat stripe rust. Previous research reported that viability of urediniospores of *M. medusa* was reduced when they were combined with conidia of *Cladosporium* spp. [19]. The percentage of conidia in spore mixtures was positively correlated with the reduction of urediniospore germination [20]. The present study also showed that urediniospore viability greatly declined when infected with *C. cladosporioides*. Hyperparasitic fungal infection can present problems in studying stripe rust. Based on our experience during the present study, maintaining low relative humidity in growth chambers during the growth of wheat plants after inoculation can reduce complications due to the hyperparasite. Specific fungicides could be tested to eliminate the hyperparasite without killing *Pst*.

For management of a hyperparasite problem, or for deployment as a biocontrol agent to control the disease on wheat, it is important to understand the interactions between the fungal host and the hyperparasite. During the present study, we observed that after inoculation with mixed spores on wheat plants, urediniospores of *Pst* appeared first on the wheat leaves, then *C. cladosporioides* started to grow. It is still not clear whether the hyperparasite infects *Pst* at germination, formation of the germ tube, formation of infection hyphae within the leaf tissue, or only during sporulation, as observed in the present study. For use as a biocontrol agent, it is also important to determine if *C. cladosporioides* does no harm to other crops and that it has any no adverse effects on humans, animals or the environment. The present study can provide the basis for such further studies.

## Materials and Methods

### Isolation and purification of the hyperparasitic fungus

In the spring of 2013, we were multiplying *Pst* urediniospores and conducting various experiments with urediniospores in our greenhouses at Northwest A&F University, Yangling, Shaanxi, China according to our commonly used methods. Seedlings of wheat were grown in the greenhouse, inoculated with normal-colored urediniospores of *Pst* at the two-leaf stage, and incubated in a dew chamber at 10°C for 24 h for multiplication. Subsequently plants were kept in a growth chamber at 13–17°C and 80–90% relative humidity [21]. About 14–20 days after inoculation, we found that uredinia on more than 45% of wheat leaves in a tray of 151 plants changed from normal fresh yellow-orange color to gray. Approximtely 20 days after inoculation with *Pst*, gray mycelia were removed from infected *Pst* uredinia with a sterilized needle and transferred to MEA medium. After incubation at 20°C for 5 days, new mycelia from the colony margins were transferred to fresh MEA medium to obtain a purified culture.

### Morphological observation

Morphological characteristics of hyphae, conidiophores and conidia of the parasitic fungus grown on MEA plates were examined for color and size, and the conidia septa changes were measured with an Olympus BX51T-32P01 optical microscope (Olympus, Japan).

To observe the ultrastructure of the parasitic fungus, wheat leaves bearing uredinia with abnormal colors were cut into 0.5×0.5 cm pieces, immersed in 4% glutaraldehyde (in 0.2 M phosphate buffer, pH 6.8) and fixed at 4°C overnight. Fixed leaf samples were washed four times with phosphate buffer for 15 min each. Thereafter, samples were successively dehydrated for 30 min each in 30, 50, 70, 80 and 90% ethanol, and finally three times in 100% ethanol. The dehydrated samples were treated with isoamyl acetate twice for 20 min each. After drying in a CO_2_ vacuum, the samples were sputter-coated with gold in an E-1045 (Hitachi, Japan) and then were examined with an S-4800 SEM (Hitachi, Japan).

### Pathogenicity test to confirm hyperparasitism

To confirm that the isolated *C. cladosporioides* strain could parasitize *Pst* urediniospores, Mingxian 169, a susceptible cultivar, was chosen for increasing urediniospores of *Pst*. When the first leaf had expanded (8–10 days, 18–20°C), plants were inoculated with CYR32,a predominant race of *Pst* in China. Fourteen days after inoculation, when infected leaves began to produce urediniospores, the diseased leaves were inoculated with the mycoparasite CGMCC 7.175. The pure culture of CGMCC 7.175 was formulated into a spore suspension (10×10 magnification field of view of about 100 spores), and the suspension was applied to uredinia on diseased leaves and on healthy wheat leaves as control check 1 (CK1). Uredinia on diseased leaves were also treated in the same manner with sterile water as CK2. For each of these three treatments, three pots of about 20 plants each were used. The inoculation with the spores, incubation in a dew chamber, incubation of the inoculated plants in a growth chamber, and observation of the symptoms and signs were performed under the same conditions described above. Meanwhile, sampling the diseased leaves inoculated with CGMCC 7.175 from 0 hpi (hours post inoculation) to 120 hpi for SEM observation, SEM treatments were the same as described above for ultrastructural observations. When symptoms appeared, the colony was removed and cultured again to confirm that it was, in fact, the mycoparasite used in the inoculations.

### Molecular characterization

#### DNA extraction

DNA was extracted from mycelium using the method described by Gams et al. [22] with modifications. To a 1.5 ml sterile centrifuge tube, 1 mg of mycelia and 200 µl of 2% CTAB [0.05M CTAB, 0.14M NaCl, 0.2M Tris-HCl (pH 8.0), 20 mM EDTA (pH 8.0)] were added. The mixture was stirred with a mini electric drill for 1 min, supplemented with another 400 µL of 2% CTAB, mixed gently, and incubated at 65°C for 2 h. After mixing with 600 µL of phenol/chloroform/isoamyl alcohol (25∶24∶1), the emulsion was centrifuged for 10 min at 15,000×g at 4°C. The aqueous phase was transferred to a fresh tube, and 600 µL of chloroform/isoamyl alcohol (24∶1) were added, mixed and centrifuged for 10 min at 15,000×g. The aqueous phase was transferred to a fresh tube and an equal volume of cold isopropanol was added. The solution was kept at −20°C overnight and centrifuged for 10 min at 15,000×g. The pellet was washed with 70% ethanol and then with 95% ethanol. After drying the DNA for 30 min in an ultra-clean hood, the DNA was dissolved in 50 µL of 1× TE buffer. Then, 1.0 µL of 20 µL/mL RNase A was added and the solution was incubated at 37°C for 30 min. DNA concentration was determined with a ND-1000 spectrophotometer (Bio-Rad, CA, USA). Purified DNA was stored at −20°C. For PCR amplification, the stock DNA solution was diluted to 100 ng/µL as the working solution and kept at 4°C.

#### PCR amplification

Partial gene sequences were determined as described by Crous et al. [23] for ITS, ACT and EF. The ITS1 (5′-TCCGTAGGTGAACCTGCG-3′) and ITS4 (5′-TCCTCCGCTTATTGATATGC-3′) [24], ACT512F (5′-TACGAGTCCTTCTGGCCCAT-3′) and ACT783R (5′-CTTGGTCATTTAGAGGAAGTAA-3′) [25] and EF446F (5′-TCACTTGATCTA CAAGTGCGGTGG-3′) and EF1035R (5′-GGTGATACC ACGCTCACGCTC-3′) [26] primers were synthesized by Biological Engineering Co. (Shanghai, China). The PCR mixtures and the amplification conditions were the same for all three genes.

The PCR mixture of 25 µL consisted of 2.5 µL of 10× *Taq* polymerase buffer (with a final concentration of 50 mM KCl, 0.1% Triton X-100, 10 mM Tris-HCl, pH 8.0); 2.0 µL of 2.5 mM of the dATP, dCTP, dGTP and dTTP mixture (TaKaRa, Japan); 2.0 µL of 25 mM MgCl_2_; 1.0 µL of 10.0 µM of each primer; 1.0 µL working solution DNA as template; 0.2 µL of 5 unit/µL *Taq* DNA polymerase (TaKaRa, Japan); and 15.3 µL of sterile double distilled H_2_O. PCR amplifications were carried out in a BIO-RAD Thermal Cycler (Bio-Rad, CA, USA). The amplification program consisted of an initial denaturation at 94°C for 2 min; 35 cycles of 94°C for 30 s, 52°C for 30 s and 72°C for 1 min; and then at 72°C for 10 min. Two µL of the PCR product was electrophoresed on a 1% agarose gel in 0.5× TBE buffer (0.089 M Tris-borate, 0.089 M boric acid and 0.002 M EDTA) for 1.5 h at 100 V. The gel was stained with ethidium bromide and bands were detected under UV light in a BIO-RAD Universal Hood II Auto Gel Documentation and Image Analysis System (BIO-RAD, CA, USA). The PCR products were purified and sequenced at the Biological Engineering Co. (Shanghai, China).

Because the morphological data indicated that the hyperparasitic fungal strain is a species within the genus *Cladosporium*, the sequences of the three genes were aligned with those of the *Cladosporium* spp. (Table 1) in GenBank using Clustal × (1.83) software [27]. Sequences were manually edited using BioEdit 5.0.9.1 [28]. A neighbor-joining tree [29] was generated based on the combined sequence data of the three genes using MEGA 6.0 [30]. The robustness of the major branches was determined with 1000 replications using bootstrap [31], [32].

**Table 1:** Accession numbers of *Cladosporium* species used in the molecular characterization.

Species	Accession Number	GenBank numbers (ITS, EF, ACT)	Substrate	Country	Reference
*C. cladosporioides*	CBS 143.35	HM148011 HM148252 HM148498	*Pisum sativum*	South Africa	[11]
*C. cladosporioides*	CBS 144.35	HM148012 HM148253 HM148499	*Pisum sativum*	U.S.A.: California	[11]
*C. cladosporioides*	CBS 145.35	HM148013 HM148254 HM148500	*Pisum sativum*	Germany	[11]
*C. cladosporioides*	CBS 101367	HM148002 HM148489 HM148243	Soil	Brazil	[11]
*C. cladosporioides*	CPC 14018	HM148050 HM148291 HM148537	*Triticum aestivum*	South Africa	[11]
*C. cladosporioides*	CPC 14244	HM148044 HM148285 HM148531	*Magnolia sp.*	U.S.A.: Louisiana	[11]
*C. pseudocladosporioides*	CBS 149.66	HM148161 HM148405 HM148650	*Triticum aestivum*	U.S.A.	[11]
*C. pseudocladosporioides*	CBS 176.82	HM148162 HM148406 HM148651	*Pteridium aquilinum*	Romania	[11]
*C. salinae*	CBS 119413	DQ780374 JN906993 EF101390	Hypersaline water	Slovenia, Sečovlje	[33]
*C. xylophilum*	CBS 126588	HM148231 HM148477 HM148722	Twigs of Salix viminalis	Italy	[11]
*C. xylophilum*	CPC 14281	HM148233 HM148479 HM148724	Leaves	France	[11]
